# First Record of *Culicoides oxystoma* Kieffer and Diversity of Species within the Schultzei Group of *Culicoides* Latreille (Diptera: Ceratopogonidae) Biting Midges in Senegal

**DOI:** 10.1371/journal.pone.0084316

**Published:** 2013-12-30

**Authors:** Mame T. Bakhoum, Moussa Fall, Assane G. Fall, Glenn A. Bellis, Yuval Gottlieb, Karien Labuschagne, Gert J. Venter, Mariame Diop, Iba Mall, Momar T. Seck, Xavier Allène, Maryam Diarra, Laëtitia Gardès, Jérémy Bouyer, Jean-Claude Delécolle, Thomas Balenghien, Claire Garros

**Affiliations:** 1 Institut Sénégalais de Recherches Agricoles, Laboratoire National de l’Elevage et de Recherches Vétérinaires, Dakar, Sénégal; 2 Department of Agriculture, Winnellie, Australia; 3 Koret School of Veterinary Medicine, Robert H. Smith Faculty of Agriculture, Food, and Environment, The Hebrew University of Jerusalem, Rehovot, Israel; 4 Parasites, Vectors and Vector-borne Diseases, Agricultural Research Council-Onderstepoort Veterinary Institute, Onderstepoort, South Africa; 5 Department of Zoology & Entomology, University of Pretoria, Hatfield, Pretoria, South Africa; 6 Department of Veterinary Tropical Diseases, University of Pretoria, Onderstepoort, South Africa; 7 Cirad, UMR15 Contrôle des Maladies; INRA, UMR1309 Contrôle des Maladies, Montpellier, France; 8 Institut de Parasitologie et de Pathologie Tropicale de Strasbourg, Faculté de Médecine, Strasbourg, France; University of California, Berkeley, United States of America

## Abstract

The Schultzei group of *Culicoides* Latreille (Diptera: Ceratopogonidae) is distributed throughout Africa to northern Asia and Australasia and includes several potential vector species of livestock pathogens. The taxonomy of the species belonging to this species group is confounded by the wide geographical distribution and morphological variation exhibited by many species. In this work, morphological and molecular approaches were combined to assess the taxonomic validity of the species and morphological variants of the Schultzei group found in Senegal by comparing their genetic diversity with that of specimens from other geographical regions. The species list for Senegal was updated with four species: *Culicoides kingi*, *C. oxystoma*, *C. enderleini* and *C. nevilli* being recorded. This is the first record of *C. oxystoma* from Africa south of Sahara, and its genetic relationship with samples from Israel, Japan and Australia is presented. This work provides a basis for ecological studies of the seasonal and spatial dynamics of species of this species group that will contribute to better understanding of the epidemiology of the viruses they transmit.

## Introduction

The recent introduction and expansion of bluetongue virus (BTV) [1], [2] and emergence of Schmallenberg virus [3] in Europe has emphasis the importance of accurate species identification and taxonomy of the genus *Culicoides* Latreille (Diptera: Ceratopogonidae) responsible for transmitting these viruses. Currently approximately 1,400 species placed into 29 formal subgenera and 39 informal species groups are recognized worldwide [4], [5]. In the Afrotropical region, some 156 species have been described but many more await description [6] and most of the literature on the taxonomy of Afrotropical species are in the need for revision [7]–[10].

The Afrotropical region is endemic for most of the known *Culicoides*-borne economical important diseases of livestock, like bluetongue virus and epizootic haemorrhagic disease virus (EHDV) [11]. While many local breeds of livestock appear to have achieved some level of tolerance to some of these diseases [12], others, for example African horse sickness virus (AHSV), still cause serious outbreaks in many parts of Africa [13] as recently illustrated with the epidemic recorded in Senegal in 2007 [14], [15].

In the Afrotropical region, *Culicoides imicola* Kieffer, subgenus *Avaritia* Fox, is regarded as the most important and proven orbivirus vector species of livestock diseases and this was recently reinforced when this species became an apparent invasive species throughout the Mediterranean basin [16], [17]. Other groupings of *Culicoides* which have been implicated in the transmission of some of these viruses is the Schultzei species group. Species belonging to this group have been associated with BTV [18], [19], AHSV [20] and EHDV [21], [22]. In especially the Australian and Oriental subregions, *C. oxystoma* Kieffer is a well-known vector of bovine arboviruses such as Akabane virus [23], [24] and is suspected of being vector of EHDV in Israel [25].

The status of the Schultzei group and its subgeneric affiliation has been disputed. Several authors placed this species group within *C.* subg. *Remmia* Glukhova [4], [6], [26] with regarding this group as a valid subgenus, while others maintain that *C.* subg. *Remmia* is a junior synonym of *C.* subg. *Oecacta* (Poey) [8], [27] or leave the group as unplaced to subgenus [28]. The status of the species within this group is equally contentious with widespread use of the name *C. schultzei* for members of the group [29]. The revision of Afrotropical species by Cornet & Brunhes [8] resolved most of the issues associated with the African fauna and Borkent [4] currently places eight species in *C.* subg. *Remmia* ( =  Schultzei group): *C. schultzei* (Enderlein), *C. oxystoma*, *C. kingi* Austen, *C. rhizophorensis* Khamala and Kettle, *C. neoschultzei* Boorman and Meiswinkel, *C. subschultzei* Cornet and Brunhes, *C. enderleini* Cornet and Brunhes and *C. nevilli* Cornet and Brunhes.

*Culicoides oxystoma* is known from the Oriental and Australasian regions [28], whereas all the other species within the Schultzei group are confined to the afrotropical region with some species extending north to the Mediterranean and Middle East [8], [18], [25], [28], [30]–[34]. The identity of the most widespread species, currently referred to as *C. oxystoma*, is unclear as many of the records listed by Wirth & Hubert [28] are based on misidentifications of *C. schultzei* and no taxonomic revisions of the Schultzei group have included this species. One barrier to the inclusion of *C. oxystoma* in a revision is that the original description by Kieffer is very brief [35] and the type specimen of *C. oxystoma* from Kolkata ( =  Calcutta) in India, has been lost [28].

**Figure 1:**
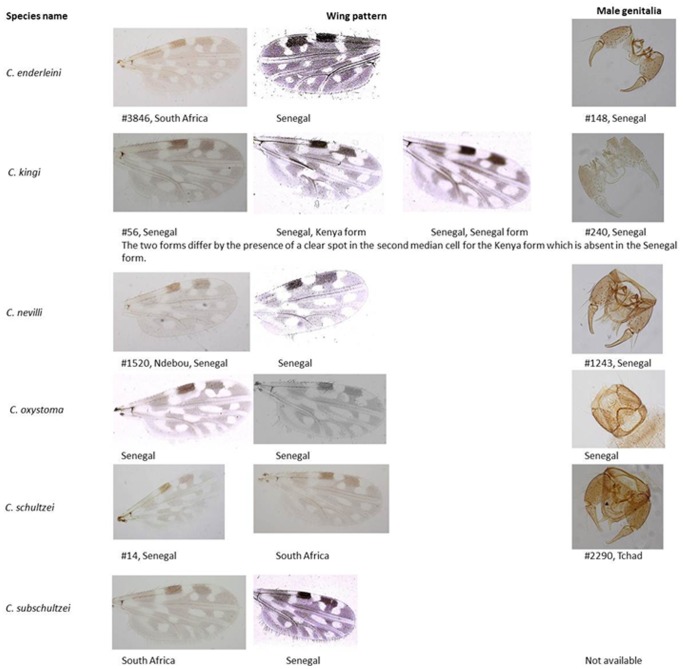
Female wing pattern and male genitalia of the Schultzei species group collected in Senegal (samples from the study and Cornet’s collection) and South Africa. The slide reference number refers to the Cornet collection reference numbers [8].

Cornet & Brunhes [8] listed three species belonging to the Schultzei group from Senegal: *C. enderleini, C. nevilli* and two morphological forms of *C. kingi* termed a Kenya form and a Senegal form. They separated these latter two forms by the degree of posterior expansion of the basal pale spot basally in cell m_1_ of the wing with this spot crossing vein M_2_ in the Kenya form but not crossing the vein in the Senegal form (Figure 1). This morphological difference appeared to be associated with a difference in breeding habitat but whether these two forms constitute a single species or not remains unclear. Similarly, the morphological similarity between Oriental *C. oxystoma* and the Afrotropical *C. subschultzei* makes differentiation of these species problematic [36]. *Culicoides schultzei* ( = *C. irroratus*) is recorded in southern, eastern and central Africa [8], although it has not been recorded in western Africa [9].

Using molecular analysis of the Cytochrome Oxidase I (COI) gene, Morag et al. [25] recently established that two distinct lineages of the Schultzei group are present in Israel. Not only does this suggest the presence of two species in Israel, one of which is synonymous with the species in Japan, it also suggests that analysis of the COI gene may be useful in addressing some of the taxonomic problems associated with this group, for example the status of the Kenya and Senegal forms of *C. kingi* and that of *C. oxystoma* and *C. subschultzei*. In this work, we combined morphological and molecular analyses to assess the diversity of the species of the Schultzei group present in Senegal in relation to that found in other regions. The specific status of morphological variants of *C. kingi* and *C. oxystoma* present in Senegal was established and an updated species list of Senegal for this group is provided.

## Materials and Methods

### Specimen Collection

**Figure 2:**
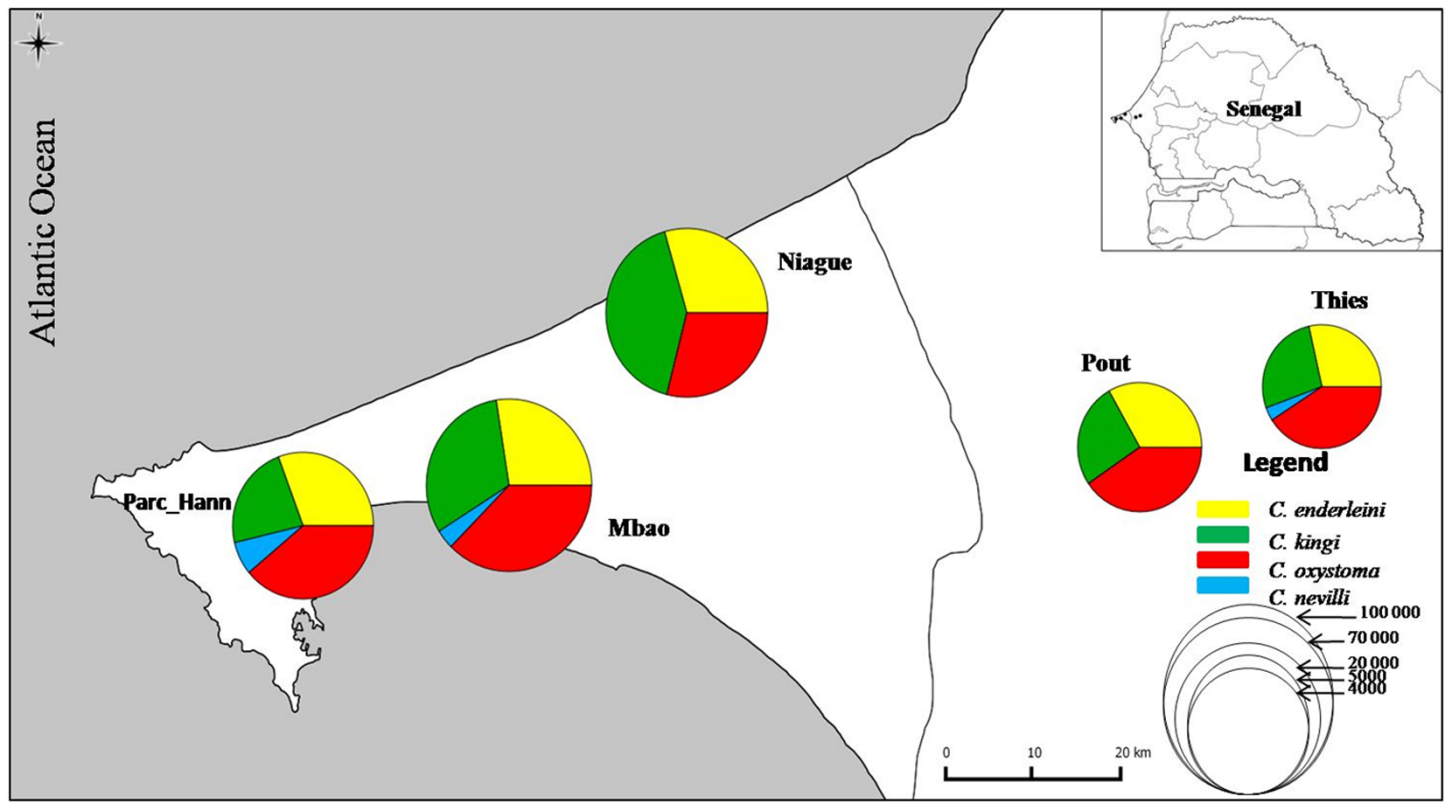
Location of the study sites in Senegal and abundance of species from the Schultzei group.

Adult midges were collected using black light/suction traps placed near horses within stud farms at five sites in Senegal (Figure 2) between September and October 2011 (3 nightly trappings/site/month). The owners of stud farms gave permission to conduct the study on their sites. Field workers did not have any contact with the horses. Specimens were preserved in 70% ethanol and identified and sexed under a binocular microscope using the identification keys of Glick [7], Cornet & Brunhes [8] and Boorman [37]. For each species, specimens representing all available morphological variants were included in the analysis. Wings from females and male genitalia were dissected prior to processing and slide-mounted to record these morphological variations. Samples from La Reunion Island (*C. enderleini*), South Africa (*C. enderleini*, *C. subschultzei, C. schultzei*), and Australia (*C. oxystoma*) were used to represent the geographic variation of these species. COI sequences submitted by Morag et al [25], Matsumoto et al [38] and Augot et al [39] were added to the dataset (Table 1).

**Table 1:** Female samples used for the molecular analysis, localization and Genbank accession numbers for the COI sequences.

Species	Geographical origin	Genbank accession numbers
*C. enderleini*	Reunion Island, France, collected in 2005	KF682426–KF682428
*C. enderleini*	Onderstepoort, South Africa, collected in 2009	KF682478–KF682479
*C. enderleini*	Madagascar, from Augot et al (2013)	KF186429–KF186431
*C. enderleini*	Senegal, collected in 2011	KF682471–KF682477
*C. kingi*	Senegal, collected in 2011	KF682482–KF682495
*C. nevilli*	Senegal, collected in 2011	KF682496–KF682497
*C. nevilli*	Madagascar, from Augot et al (2013)	KF186428
*C. oxystoma*	Senegal, collected in 2011	KF682498–KF682522
C. *oxystoma*	Australia	KF682529–KF682533
*C. oxystoma*	Israel, from Morag et al (2012)	JN545045–JN545049, JN545052, JN545054
Schultzei Group	Israel, from Morag et al (2012)	JN545050–JN545051, JN545053
*C. oxystoma*	Japan, from Matsumoto et al (2009)	AB360978, AB360980–AB360985
*C. subschultzei*	Skukuza Kinger national park, South Africa, collected in 2008	KF682523–KF682525
*C. imicola*	Reunion Island, France, collected in 2005	KF642480–KF682481

### Extraction of Genomic DNA

Genomic DNA was individually extracted following an extraction protocol with Chelex resin in 5% (Resin Chelex100 ®, Chelating Ion Exchange Resin, Bio-Rad, France ) as described in Viennet et al [40] and Solano et al [41]. A volume of 500 µl of Chelex solution was dispensed into each tube of 1.5 ml. Each individual was removed from ethanol and dried on blotting paper. The individual was retrieved and transferred to a tube with Chelex solution and ground using a piston. The tubes were incubated at 56°C for 60 minutes and then at 95°C for 30 minutes (for thermal lysis). Immediately after heating, the tubes were centrifuged at 13,000 revs/min for 1 minute to pellet the Chelex resin with inhibitor ions and cellular debris.

### Polymerase Chain Reaction Amplification and Sequencing of COI

Cytochrome Oxidase I (COI) amplification of the gene was carried out using primers C1J1718 (Forward) 5′- GGA- GGA-TTT-GGA-AAT-TGA-ATT-GT-3′ and C1N2191 (Reverse) 5′ -CAG-GTA-TTA-AAA-AAA-AAA-TAT-CTT-CTG-G-3′ to obtain an approximately 600 bp product as described previously [42]. Amplification reactions by Polymerase Chain Reaction (PCR) were performed in 25 µl of reaction volume with 5 µl of buffer 5X, 0.5 µl of dNTP (10 mM), 2 µl of MgCl2 (25 mM), 0.5 ml of each primer; 0.5 µl of Taq polymerase (5 U/µl), 15 µl of sterile water and 1 µl of DNA. Touch-up PCR amplification was used to reduce non-specific amplifications and optimize the quality of amplification for better sequencing. The cycling profile of the COI gene consisted of an initial denaturation stage of 1 minute at 94°C, followed by 5 cycles of 40 seconds at 94°C, 40 seconds at 45°C and 1 minute at 72°C, then 35 cycles of 40 seconds at 94°C, 40 seconds at 51°C, 1 minute at 72°C and a final elongation of 5 minutes at 72°C. The PCR products were visualized on 1% agarose gel with a Gel Red staining after migration of 90 minutes at 100 volts by electrophoresis, before being sent to Cogenics (Grenoble, France) for sequencing.

### Sequence Alignment, Phylogenetic and Genetic Distance Analyses

Multiple alignments of the sequences were generated using the CLUSTALW algorithm in BioEdit [43]. Molecular evolutionary analyses were conducted using DAMBE [44] and MEGA version 5 [45]. The phylogenetic reconstructions were performed by Maximum Likelihood (ML) and Bayesian analyses (BA). The ML analyses were carried out with MEGA v5 [45], incorporating best fit models of sequence evolution determined using the Akaike Information Criterion (AIC) and employing 1,000 bootstrap replications to determine node reliabilities. The AIC implemented within jModelTest was used to determine the most suitable evolutionary model(s) for the Bayesian and ML analyses [46]. The AIC model selected for the COI was T92+G, followed by T92+G+I and HKY+G. The best fit model (T92+G) was used for both ML and BA analysis. Summary sequence statistics were generated using MEGA v5. Bayesian analyses was performed using MrBayes [47] with the following settings: the ML model employed two substitution types (“nst = 2”), with rate variation across sites modeled using a gamma distribution (rates = ”gamma”); Markov Chain Monte Carlo searches were done with four chains for 500,000 generations, with trees sampled every 100 generations (the first 1,000 trees were discarded as “burn in”). The appropriate burn-in fraction and convergence of the Markov Chain Monte Carlo chains were graphically assessed by evaluating the stationary phase of the chains using Tracer v1.5 [48]. Convergence metrics provided by MrBayes were checked to ensure that the maximum standard deviation of split frequencies of any of the runs was under 0.05 and that the potential scale reduction factor for all parameters approached 1.0. *Culicoides imicola* was used as an out-group. Estimates of average evolutionary divergence over sequence pairs within and between groups were made using the Maximum Composite Likelihood model for the COI sequences. The average genetic distances between the clades inferred by phylogenetic analyses were computed by Tamura-3 parameter or Jukes-Cantor model with the program Mega v5. The two models gave the same genetic distance matrix (data shown for JC model).

## Results

### Morphological Identification

**Figure 3:**
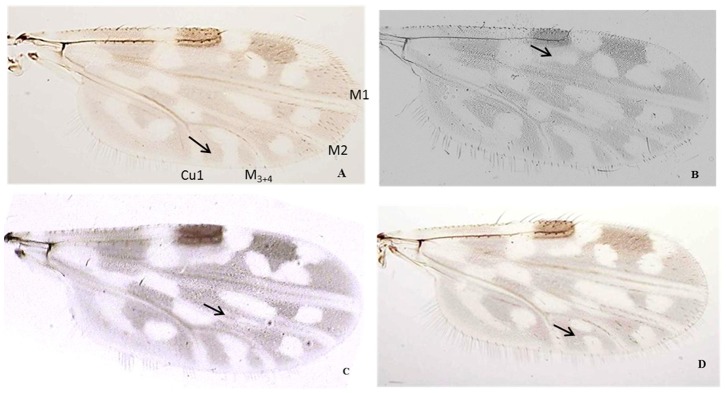
Variation in the wing patterns of *C. subschultzei* and *C. oxystoma* found in Senegal: *Culicoides subschultzei* typical form (A), *Culicoides oxystoma* typical form (B) and morphological variants: absence of pale spots in the second median cell (C) and presence of two pale spots in cubital cell (D).

Morphological examination of 82,506 individuals from the 5 study sites revealed the presence of four species belonging to the Schultzei group (Figure 2). These species were referable to *C. kingi* (both the Kenya and Senegal forms sensu Cornet & Bruhnes), *C. oxystoma* (showing large phenotypic variation) (Figure 3), *C. enderleini* and *C. nevilli*. Although *C. nevilli* was present at three sites, the other species were abundant and equally present on the five sampled sites.

### Phylogenetic Analysis

**Figure 4:**
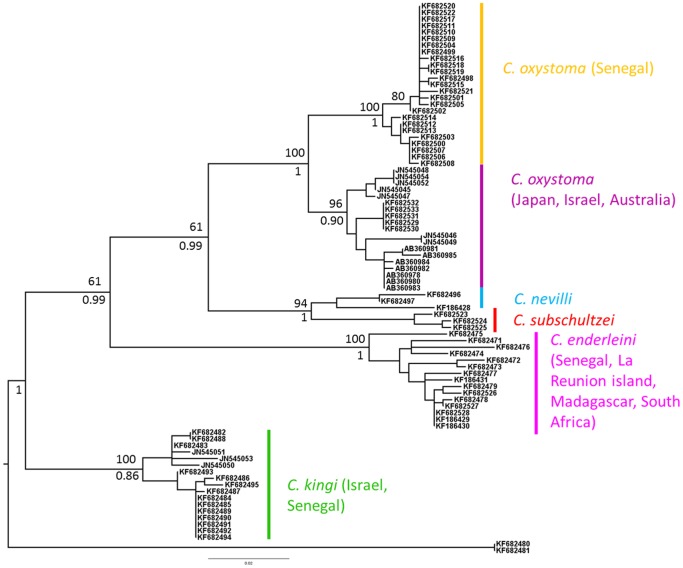
Phylogenetic analysis of species of the Schultzei group using COI sequence data. The topology shows Bayesian inference tree using program MrBayes. *Culicoides imicola* was designated as an outgroup taxon. Numbers indicate bootstrap values from ML (bottom) analyses, and posterior probabilities from Bayesian analysis (top). Only bootstrap values >60% and Bayesian posterior probabilities >0.5 are shown. Branch lengths represent nucleotide substitutions per site.

A total of 63 COI sequences were obtained referable to six species including *C. imicola* used as out-group (Table 1). Twenty COI sequences were added to the dataset from the literature (Table 1). Sequencing of COI sequences of *C. schultzei* was not successful. Four individuals of each *C. kingi* form were obtained (Kenya form: KF682484–KF682485, KF682491–KF682492); Senegal form: KF682487, KF682489–KF682490, KF682494). The ML and BA trees depicted the same topology. Six distinct and well-supported phylogenetic lineages were clearly differentiated (Figure 4). One clade grouped the individuals identified as *C. kingi*, including both the Senegal and Kenya forms, and the three unidentified sequences from Israel (JN545050–51, JN545053). Another included all specimens of *C. enderleini* from all different location sites. Specimens of *C. oxystoma* formed two separate clades, one including all specimens from the Palaearctic and Australasian regions (Japan, Israel, and Australia) and the other including all individuals from Senegal. The mean genetic distance between these two clades was two times higher than the mean variation within a clade. The specimens from Senegal identified as *C. oxystoma* and representing all of the morphological variations illustrated in Figure 2 were clustered in the same clade. The maximum variation within a clade was 2.8% within the *C. nevilli* clade, and the minimum distance between clades being 1.5% between *C. oxystoma* from Australia and Japan (Table 2).

**Table 2:** Estimates of evolutionary divergence of sequence pairs between and within populations and species.

	Within species/groupmean distance	*C. enderleini*	*C. kingi*	*C. nevilli*	*C. oxystoma* Australia	*C. oxystoma* Israel	*C. oxystoma* Japan	*C. oxystoma* Senegal
***C. enderleini***	0.022	–	–	–	–	–	–	–
***C. kingi***	0.015	0.0132	–	–	–	–	–	–
***C. nevilli***	0.028	0.12	0.106	–	–	–	–	–
***C. oxystoma*** ** Australia**	0.000	0.125	0.102	0.07	–	–	–	–
***C. oxystoma*** ** Israel**	0.015	0.131	0.103	0.079	0.019	–	–	–
***C. oxystoma*** ** Japan**	0.005	0.124	0.101	0.073	0.015	0.021	–	–
***C. oxystoma*** ** Senegal**	0.009	0.131	0.098	0.09	0.041	0.046	0.043	–
***C. subschultzei***	0.011	0.119	0.112	0.054	0.082	0.092	0.091	0.1

Analyses were conducted using the Jukes-Cantor model. The analysis involved 82 nucleotide sequences. Codon positions included were 1st+2nd+3rd+Noncoding. All positions containing gaps and missing data were eliminated. There were a total of 472 positions in the final dataset.

## Discussion

At least four species belonging to the Schultzei group are now known from Senegal: *C. enderleini*, *C. kingi*, *C. nevilli*, and *C. oxystoma*. Our phylogenetic analyses have identified the three unidentified individuals (JN545050–51, JN545053) from Morag et al. (2012) as *C. kingi* which confirms the presence of this species in Israel. Our study confirmed that the Kenya and Senegal forms of this species, described by Cornet & Brunhes (1994) partially based on differing larval ecology, do not appear to be distinct species.

The relatively high genetic diversity observed within *C. nevilli* and *C. enderleini* probably reflects historically independent populations of these species on Madagascar and on mainland Africa as recently observed within Australian populations of *C. immaculatus* [49]. Overall, the *Culicoides* fauna of Madagascar has been understudied [39], [50] and remains to be investigated.

The genetic similarity between Oriental and Australian specimens of *C. oxystoma* and those from Senegal confirms the identity of this species and is the first record of *C. oxystoma* in Africa south of Sahara. There has been a great deal of confusion in the literature between this species, *C. schultzei* and *C. subschultzei* [8], [25], [37]. Boorman (1989) suggested that most of the records of *C*. *schultzei* or the Schultzei group from north of the Sahara and eastwards through India refer to *C. oxystoma* and its presence in Senegal indicates that it is also present south of the Sahara. Taken together, Boorman et al (1989) and our results showed that multiple species of the Schultzei group are sympatric in the Middle East (for instance at least *C. kingi* and *C. oxystoma* in Israel), and we recommend use of the most updated keys (Boorman 1989; Cornet and Brunhes 1994) when identifying material collecting from this area.

*Culicoides oxystoma* is a widespread species with a wide phenotypic variation that warrants further exploration. Specimens exhibiting the full range of morphological variation observed in Senegal (Figure 3) were shown to be conspecific but also showed strong support for two lineages within *C. oxystoma* indicating some level of reproductive isolation within this species. This might confirm what Boorman suspected as a differentiation within *C. oxystoma*, creating an Afrotropical lineage and a Palearctic and oriental lineage [36]. Despite the morphological similarity between *C. oxystoma* and *C. subschultzei* [36], COI analysis indicates that these two species are distinct with *C. subschultzei* being more closely related to *C. nevilli* than to *C. oxystoma*. Further investigations need to be implemented including *C. schultzei*. Unfortunately, attempts to amplify DNA from the *C. schultzei* samples from South Africa were unsuccessful, probably because they were too old (collected in 1996). Further works will also need to include other known members of the group, *C. rhizophorensis* and *C. neoschultzei*, and probably look at other species showing morphological similarity [51].

Up to date no phylogenetic study has ever successfully validated the subgeneric classification of the genus *Culicoides* [4], [5] and the validity of the Schultzei group, *C.* subg. *Remmia, C.* subg. *Oecacta*, and many other subgeneric groupings of *Culicoides,* remains doubtful. Augot et al. (2013) recently analysed the COI gene to test the monophyly of the Schultzei group and *C.* subg. *Oecacta* but their analysis were confounded by homoplasy of this gene and no conclusions could be drawn. They suggested the use of alternative genes to explore these relationships and it is likely that an integrated approach using a combination of morphological and molecular analyses might clarify this situation.

The wide distribution and economic importance of species of the Schultzei group highlight the need to re-evaluate the status of these species and a molecular approach might be an appropriate means of achieving that goal. Based on the numbers collected and wide distribution of the suspected vector species based on previous virus studies, these species can indeed play an important role in the epidemiology of AHSV in Senegal. In particular, future studies should focus on the differences between African and Oriental populations of *C. oxystoma* and establish the morphological and genetic limits of this species. Inclusion of material from the type locality of *C. oxystoma* in India would help to confirm the identity of specimens currently referred to this species.
